# Device associated nosocomial infections in a medical intensive care unit of a tertiary care hospital in Jaipur, India

**DOI:** 10.1186/1753-6561-5-S6-O16

**Published:** 2011-06-29

**Authors:** S Sood, SH Joad, D Yaduvanshi, P Anand

**Affiliations:** 1 Super Religare Laboratories, Fortis Escorts Hospital, Jaipur, India; 2 Critical Care Medicine, Fortis Escorts Hospital, Jaipur, India; 3 Pulmonary Medicine, Fortis Escorts Hospital, Jaipur, India

## Introduction / objectives

Intensive care units (ICUs) are unfortunately the epicenters of nosocomial infections. Limited data is available regarding burden of healthcare associated infections (HAIs) in Indian ICUs, especially the rates of device associated infections by using standardized definitions.

## Methods

We conducted a prospective surveillance of device associated infections from January 2010-December 2010 in a 10 beded Medical ICU of Fortis Escorts Hospital, Jaipur. CDC-NNIS system definitions for all device associated infections were used and rates were calculated per 1000 device days. Device utilization ratio was calculated by dividing the total number of specific device days by the total number of patient days. Microbiological profile of each HAI was noted.

## Results

435 patients were admitted in the Medical ICU representing 3080 patient days. The overall DANI (device associated nosocomial infection) rate was 4.36% (19/435) or 6.16 (19/3080) DAI per 1000 ICU days. The overall VAP rate was 8.9 infections per 1000 ventilator days, CLABSI rate was 2.74 infections per 1000 central day and CAUTI rate was 1.50 infections per 1000 catheter days. Device utilization ratio for central line, ventilator and urinary catheter was 0.59, 0.36 and 0.86 respectively. Non fermenters Gram negative bacteria accounted for 73.68% infections followed by Enterobacteriaceae (21.05%). The most common bacteria were *Acinetobacter baumannii* (26.31% of total) and *Pseudomonas aeruginosa* and *Klebseilla pneumoniae* (10.52% of total each).

## Conclusion

Targeted surveillance and calculation of device associated infection rates per 1000 device days allows detection of unique institutional problems that need redress.

## Disclosure of interest

None declared.

